# High leukocyte mitochondrial DNA copy number contributes to poor prognosis in breast cancer patients

**DOI:** 10.1186/s12885-023-10838-x

**Published:** 2023-04-25

**Authors:** Wenzhe Zhang, Songping Lin, Bangwei Zeng, Xiaobin Chen, Lili Chen, Minyan Chen, Wenhui Guo, Yuxiang Lin, Liuwen Yu, Jialin Hou, Yan Li, Shengmei Li, Xuan Jin, Weifeng Cai, Kun Zhang, Qian Nie, Hanxi Chen, Jing Li, Peng He, Qindong Cai, Yibin Qiu, Chuan Wang, Fangmeng Fu

**Affiliations:** 1 Department of Breast Surgery, Fujian Medical University Union Hospital, No.29, Xin Quan Road, Gulou District, Fuzhou, 350001 Fujian Province China; 2 Department of General Surgery, Fujian Medical University Union Hospital, Fuzhou, 350001 Fujian Province China; 3 Breast Cancer Institute, Fujian Medical University, Fuzhou, 350001 Fujian Province China; 4 Nosocomial Infection Control Branch, Fujian Medical University Union Hospital, Fuzhou, Fujian Province China; 5 Quanzhou First Hospital Affiliated to Fujian Medical University, Quanzhou, 362000 Fujian Province China

**Keywords:** Breast cancer, Mitochondrial DNA copy number, Leukocyte, Survival

## Abstract

**Background:**

Compelling evidence has indicated a significant association between leukocyte mitochondrial DNA copy number (mtDNAcn) and prognosis of several malignancies in a cancer-specific manner. However, whether leukocyte mtDNAcn can predict the clinical outcome of breast cancer (BC) patients has not been well investigated.

**Methods:**

The mtDNA copy number of peripheral blood leukocytes from 661 BC patients was measured using a Multiplex AccuCopy™Kit based on a multiplex fluorescence competitive PCR principle. Kaplan–Meier curves and Cox proportional hazards regression model were applied to investigate the association of mtDNAcn with invasive disease-free survival (iDFS), distant disease-free survival (DDFS), breast cancer special survival (BCSS), and overall survival (OS) of patients. The possible mtDNAcn-environment interactions were also evaluated by the Cox proportional hazard regression models.

**Results:**

BC patients with higher leukocyte mtDNA-CN exhibited a significantly worse iDFS than those with lower leukocyte mtDNAcn (5-year iDFS: fully-adjusted model: HR = 1.433[95%CI 1.038–1.978], *P* = 0.028). Interaction analyses showed that mtDNAcn was significantly associated with hormone receptor status (adjusted p for interaction: 5-year BCSS: 0.028, 5-year OS: 0.022), so further analysis was mainly in the HR subgroup. Multivariate Cox regression analysis demonstrated that mtDNAcn was an independent prognostic factor for both BCSS and OS in HR-positive patients (HR+: 5-year BCSS: adjusted HR (aHR) = 2.340[95% CI 1.163–4.708], *P* = 0.017 and 5-year OS: aHR = 2.446 [95% CI 1.218–4.913], *P* = 0.011).

**Conclusions:**

For the first time, our study demonstrated that leukocyte mtDNA copy number might influence the outcome of early-stage breast cancer patients depending on intrinsic tumor subtypes in Chinese women.

**Supplementary Information:**

The online version contains supplementary material available at 10.1186/s12885-023-10838-x.

## Introduction

Breast cancer (BC) is the first common malignancy and the fifth leading cause of cancer death among women in China [1]. BC is highly heterogeneous at molecular and clinical levels [2]. Generally, some prognostic factors for early-stage breast cancer (EBC) survival include tumor size, lymph node involvement, tumor grade, and hormone receptor (HR) status. The survival rate has increased due to improved management strategies in the past few decades [3]. However, EBC patients with the same traditional stage often show different clinical outcomes because of tumor heterogeneity.

Consequently, biomarkers are urgently needed to complement the traditional system for a more accurate prognostic prediction of EBC and thus benefit individualized treatment. Some theories indicate that genetic background is an important determinant of metastatic potential. The theories also suggest that it might be possible to define metastasis susceptibility based on gene variation in readily accessible tissues (for example, blood) rather than tumors [4]. The study of mtDNA copy number variation in breast cancer is of particular interest, given the role of oxidative stress in the disease’s etiology.

Mitochondria are essential cellular organelles in eukaryotic cells and perform multiple cellular functions, notably in energy metabolism, reactive oxygen species (ROS) generation, and apoptosis [5]. Mitochondria contain their DNA (mtDNA), which is susceptible to damage caused by high reactive oxygen species due to the lack of protective histones and its diminished DNA repair capacity [6]. There can be several 100 or even more than 1000 copies of mitochondrial genomes in each eukaryotic cell. Although mtDNA copy number is significantly varied among cells from different tissue origins [7], it has been observed that there is a good correlation between the number of mtDNA in various cell types within the same individual [8]. The mtDNA copy number may change significantly under different internal or external microenvironments, damaging the OXPHOS system and the enhanced ROS production. This scenario has been believed to contribute to the initiation and development of tumors [9].

Quantitative changes in mtDNA content have been observed in a variety of malignant tumors, such as BC [10], gastric cancer [11], head and neck cancer [12] and colorectal cancer [13]. Previous studies have also shown that changes in mtDNA content in tumor tissues were related to tumor staging, prognosis, and treatment response, again in a cancer type-specific manner [14, 15]. Yu et al. have found that reduced mitochondrial DNA copy number in tumor tissues was correlated with tumor progression and prognosis in Chinese BC patients [10]. There have been a series of reports on the relationship between mtDNA content in peripheral blood leukocytes and cancer susceptibility [16–19]. However, only a few studies have shown that higher mtDNA content in peripheral blood leukocytes is associated with poor prognosis in colorectal cancer [20], hepatocellular carcinoma [21], and glioma [22]. In addition, Xia et al. have found that leukocyte mtDNA content is associated with the T stage of BC, indicating that high mtDNA content may facilitate cancer progression, but did not explore the relationship with the prognosis of BC [23]. Thus, the effect of mtDNA copy number in peripheral blood leukocytes on BC patient prognosis has not been explored.

This study measured the leukocyte mtDNA copy number in peripheral blood from EBC patients and analyzed the association between leukocyte mtDNA copy number and the survival of patients in Southeastern China with EBC. To the best of our knowledge, this is the first study to assess mtDNA content’s value in predicting the prognosis of EBC patients.

## Materials and methods

### Study population

Our hospital-based study recruited 681 BC cases from Fujian Medical University Union Hospital between July 2000 and October 2014. Patient eligibility criteria in this study were as follows: (i) histopathologically confirmed with invasive BC; (ii) subsequently treated with curative surgical resection and systemic therapy; (iii) availability of complete clinical and follow-up data, (iv) no history of other malignancy and (v) alive at least 1 month after surgery. According to this screening criteria, we ruled out 20 patients, containing 6 patients with breast carcinoma in situ and 14 patients with incomplete clinical information. Finally, 661 patients with resected BC were included in the present study for predictive analysis.

Clinicopathological and demographic information was collected from the hospital records, and survival data were obtained from the followed-up database renewed annually. The patients were staged according to the 7th version of the American Joint Commission on Cancer (AJCC) tumor-node-metastasis (TNM) staging system [24]. Estrogen receptor (ER)/progesterone receptor (PR) positivity was determined by IHC analysis of the number of positively stained nuclei (≥ 10%), and HR positivity was defined as being either ER + and/or PR+. Tumors were considered human epidermal growth factor-2 (HER2) positive when cells exhibited strong membrane staining (3+). Expressions of 2 + would require further in situ hybridization testing for HER2 gene amplification, while expressions of 0 or 1 + were regarded as negative. The subtypes were categorized [25]: luminal A (ER+, PR+ >20%, HER2-, Ki67<14% or grade I when Ki67 was unavailable), luminal B (HR+, HER2-, Ki67>14% or grade II/III when Ki67 was unavailable or HR+, HER2+); HER2 enriched (HR-, HER2+) and triple-negative (HR- and HER2-). The Institutional Ethics Committee approved the study, and all participants consented to the testing at the time of their participation and contributed data.

### DNA extraction

Blood samples were collected in EDTA anticoagulant tubes and were centrifuged within 30 min. Genomic DNA was extracted from blood samples by using the Whole-Blood DNA Extraction Kit (Bioteke, Beijing, China), according to the manufacturer’s protocol, and then the genomic DNA was aliquoted and stored at -80 °C for future analysis. The purity of the DNA samples was assessed before mtDNA copy number assessment, and samples without sufficient DNA yield were not included in mtDNA copy number analysis.

### MtDNA copy number assessment

Relative mtDNA content was measured by a custom-by-design Multiplex AccuCopy™Kit (Genesky Biotechnologies Inc., Shanghai, China) based on a multiplex fluorescence competitive PCR principle as previously described [26] which can interrogate copy number variation (CNV) status at multiple genomic loci in the same assay reaction.

The methods below briefly describe the manufacturer’s process. A total of 2 target genomic segments within the ND1 gene and 6 reference segments (2, 10, 16, 18, 19, and 20 p) were chosen for the AccuCopy assay. The primers for both target and reference segments were synthesized. The forward primers were fluorescent-labeled at Genesky Biotechnologies (Shanghai, China). The competitive DNAs for the ND1 and 6 reference segments were synthesized in double-strand and provided a mixture from Genesky Biotechnologies (Shanghai, China). These competitive DNAs are almost identical to their homologies in the human reference genome except for 1–2 base pairs deleted. The primers of target segments, reference segments, and probes information were shown in Table S1.

The PCR reaction was prepared in 20 µl for each sample, containing a mixture of 2 µl target genomic DNA (5 ng/µl) with 2 µl reference segment DNA, 1 µl Multiplex PCR Fluorescence Primer Mix (AccuCopy™), 10 µl 2 × PCR Master Mix (Genesky Biotechnologies), and 5 µl ddH2O. 10 µl 2 × PCR Master Mix (Genesky Biotechnologies), and 5 µl ddH2O. The program used was an initial denaturation step of 95 °C for 10 min followed by the first 11 cycles of denaturation at 94 °C for 20 s, annealing at 60 °C for 40 s (the annealing temperature was decreased by 0.5 °C in each consecutive cycle), and elongation at 72 °C for 1.5 min, followed by the second 24 cycles of denaturation at 94 °C for 20 s, annealing at 59 °C for 30 s, and elongation at 72 °C for 1.5 min, and a final extension step at 60 °C for 60 min and then at 4 °C forever. The PCR product was diluted in a 1:5 ratio, and 1 µl diluted products were mixed with 0.5 µl 500 (Liz) size standard and 8.5 µl Hi-Di formamide (both from Applied Biosystems, Foster City, CA). The mixture was subjected to a denaturation step of 95 °C for 5 min and electrophoresed in a 3730XL genetic analyzer (ABI, Carlsbad, CA, USA). Raw data were analyzed by GeneMapper 4.0 (ABI). The height and area data for all specific peaks were exported into a Microsoft Excel file.

The sample/competitive (S/C) peak ratio was calculated for the 2 target and 6 reference segments. The S/C ratio for each target fragment was first normalized based on four reference segments, respectively. The 6 normalized S/C ratios were further normalized to the median value in all samples for each reference segment, respectively, and then averaged. If one of the six normalized S/C ratios deviated >25% from the average of the other five, it was excluded from further analysis. The copy number of each target segment was determined by the average S/C ratio times two, given that the copy numbers of four reference segments are two in the diploid genome. Twenty samples were randomly selected as blinded duplicates for quality assessment purposes, and excellent concordance was obtained.

### Statistical analyses

Overall survival (OS) and BC-specific survival (BCSS) were our primary endpoints and defined as the time from cancer diagnosis to mortality for all causes and BC, respectively. Invasive disease-free survival (iDFS) and distant disease-free survival (DDFS) were our secondary endpoints and calculated separately from the date of diagnosis to the date of any recurrence and distant recurrence to follow-up cut-off time [27]. Survival data were analyzed using the Kaplan–Meier method with the log-rank test. Survival analysis was discontinued at 5 years of follow-up because 5-year survival is an important prognostic indicator for BC and the survival data after five years is not yet mature. To adjust for possible confounders and evaluate the form of the relationship, Cox proportional hazards models were fitted using different adjustment parameters: model 1 adjusted for age at diagnosis; model 2 additionally adjusted for hormone receptor status and HER2 status; model 3 was the same as model 2 plus an adjustment for tumor size, Lymph node involvement, and grade. The Cox proportional hazards model results showed the non-linearity in the effects of mitochondrial copy number on cancer progression.

Moreover, nonlinear p-splines with knots were created to evaluate the functional form of the relationship between mtDNA copy number and BC patients’ survival as previously described [28, 29]. The splines’ knots were at the quintiles of the mtDNA copy number distribution. The results of the splines and the estimates for quintiles 2, 3, 4, and 5 compared with quintile 1 revealed that a categorical analysis of the data by combining the 2nd, 3rd, 4th and 5th quintile of mtDNA copy number and set quintile 1 as a reference fits the data better than modeling the data on a continuous scale. The Chi-square test was used to examine differences in categorical variables between subgroups. The student’s t-test was used to analyze the difference of normally distributed continuous variables between two groups. The Cox regression model calculated the hazard ratios (HRs) and 95% confidence interval (CI) for each factor in multivariate analyses. The main analysis started with a full Cox regression model (including copy number and all prognostic factors). A global likelihood ratio test was carried out to test whether all factors together significantly influenced BC survival. In case of a nonsignificant result, no further analyses were carried out, to avoid false-positive results. If, however, the *P*-value was significant, mtDNA copy number and all prognostic factors will be further analyzed. A stratified multivariate analysis of clinical parameters was performed to evaluate the impact of mtDNA copy number on BC prognosis in specific patients. The possible mtDNAcn-environment interactions were also evaluated by the Cox proportional hazard regression models. All tests were 2-sided, and *P*-values < 0.05 were considered statistically significant. SAS 9.4 (SAS Institute Inc., Cary, NC) was used for all statistical analyses.

## Results

### Patient characteristics and clinical features

Participants in the 661 early BC cohort were female, and their mean age was 46.73 ± 10.28 years old at BC diagnosis. The clinical characteristics and survival of the 661 BC patients are summarized in Table 1. During a follow-up time of 5 years, 279 cases experienced recurrence (61 locoregional and 254 distant), and 180 died (176 died of BC and 4 died of another disease). No significant difference in BC-iDFS, BC-DDFS, BCSS, and OS was shown in the subgroup of age at diagnosis 40 (*P* = 0.136, 0.331, 0.754 and 0.934). Nevertheless, patients with a tumor size > 2 cm, lymph node-positive, grade III, clinical-stage II + III, or HER2 positive had significantly shorter survival times, whereas HR positivity remarkably improved the survival of BC patients (log-rank *P* < 0.05, Table 1). Furthermore, our intrinsic molecular subtypes (luminal A, luminal B, HER2-enriched, and triple-negative) were also associated with significantly different survival (log-rank *P* < 0.05, Table 1). Treatment characteristics of patients showed in Table S3.

**Table 1:** Patients’ clinicopathological characteristics and clinical outcome

Variables	Patients	5-Year iDFS		5-Year DDFS		5-Year BCSS		5-Year OS	
	N = 661	Events	LogRank *P*	Events	LogRank *P*	Events	LogRank *P*	Events	LogRank *P*
Age at diagnosis			0.136		0.331		0.754		0.934
≤ 40	207	97		86		56		56	
>40	454	182		168		120		124	
Tumor size(cm)			<0.001		<0.001		<0.001		<0.001
≤ 2	211	55		48		34		34	
>2	450	224		206		142		146	
Nodal status			<0.001		<0.001		<0.001		<0.001
Negative	255	66		55		30		33	
Positive	406	213		199		146		147	
Clinical stage			<0.001		<0.001		<0.001		<0.001
I	127	21		17		12		12	
II + III	534	258		237		164		168	
Grade			<0.001		<0.001		<0.001		<0.001
I + II	500	190		172		118		121	
III	161	89		82		58		59	
HR			<0.001		<0.001		<0.001		<0.001
Negative	235	129		121		99		100	
Positive	426	150		133		77		80	
HER2			<0.001		<0.001		<0.001		<0.001
Negative	467	170		152		103		106	
Positive	194	109		102		73		74	
Subtype			<0.001		<0.001		<0.001		<0.001
LuminalA	123	18		17		8		8	
Luminal B	303	132		116		69		72	
HER2+	101	64		59		48		48	
Triple negative	134	65		62		51		52	

Abbreviations: iDFS, invasive disease-free survival; DDFS, distant disease-free survival; BCSS, breast cancer special survival; OS, overall survival; HR, Hormone receptor; HER2, human epidermal growth factor-2

### Effects of mtDNA copy number on survival of BC

**Fig. 1:**
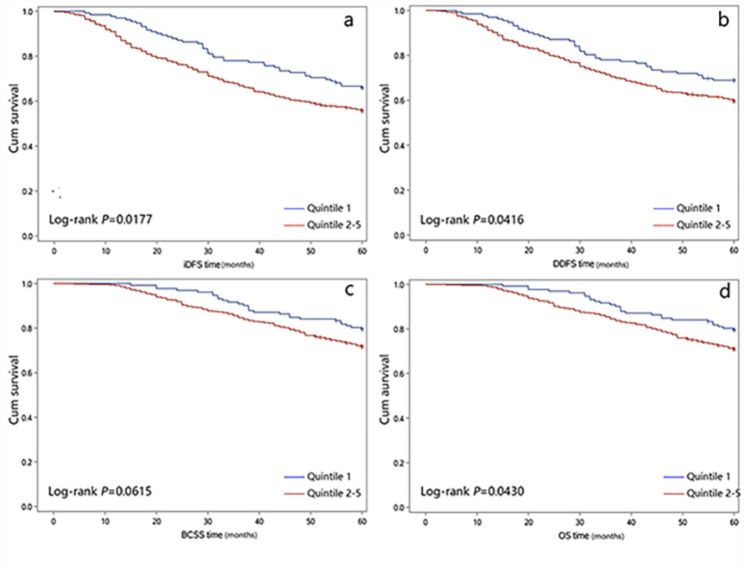
Kaplan-Meier curves for iDFS (a), DDFS (b), BCSS (c), and OS (d) due to BC by Quintile 2–5 AND Quintile 1 of mitochondrial DNA copy number. Abbreviations: iDFS, invasive disease-free survival; DDFS, distant disease-free survival; BCSS, BC special survival; OS, overall survival

We performed a Cox regression analysis based on quintile categorization of mtDNA copy numbers applying different adjustments for confounders. Since the estimates for quintiles 2, 3, 4, and 5 pointed in the same direction with no clear indication of a linear association, we set the first quintile as the reference category to illustrate the quintiles 2–5 values’ influence. Details for each quintile and adjustment model can be found in Supplementary Table S2. In line with this observation, nonlinear splines showed an increased risk for patients within the 2–5 quintiles of mtDNA copies, compared with quintile 1 for all 4 outcome variables (Figure S1). For reasons of statistical power, we collapsed quintiles 2–5 and compared them to quintile 1, which was a reference in all subsequent analyses. The detailed characteristics comparing combined quintiles 2–5 against quintile 1 showed in Table 2. Patients with higher copy numbers (quintiles 2–5) have higher grades (*P* = 0.041). The Kaplan-Meier curves and log-rank tests comparing combined quintiles 2–5 against quintile 1 (Fig. 1) showed significant differences concerning 5-year iDFS, DDFS, and OS, except for BCSS (*P* = 0.018 for iDFS, *P* = 0.042 for DDFS, *P* = 0.061 for BCSS, and *P* = 0.043 for OS).

**Table 2:** Associations between mtDNA copy number and clinicopathological characteristics in BC patients

Characteristics	Quintile 1(n = 132)	Quintile 2–5(n = 529)	*P*-value
Age at diagnosis			0.753
≤ 40	43 (32.6%)	164 (31.0%)	
>40	89 (67.4%)	365 (69.0%)	
Tumor size(cm)			0.348
≤ 2	47 (35.6%)	164 (31.0%)	
>2	85 (64.4%)	365 (69.0%)	
Nodal status			0.272
Negative	45 (34.1%)	210 (39.7%)	
Positive	87 (65.9%)	319 (60.3%)	
Clinical stage			0.622
I	23 (17.4%)	104 (19.7%)	
II + III	109 (82.6%)	425 (80.3%)	
Grade			0.041
I + II	109 (82.6%)	391 (73.9%)	
III	23 (17.4%)	138 (26.1%)	
HR			0.186
Negative	40 (30.3%)	195 (36.9%)	
Positive	92 (69.7%)	334 (63.1%)	
HER2			0.749
Negative	95 (72.0%)	372 (70.3%)	
Positive	37 (28.0%)	157 (29.7%)	
Subtype			0.393
Luminal A	28 (21.2%)	95 (18.0%)	
Luminal B	64 (48.4%)	239 (45.2%)	
HER2+	20 (15.2%)	81 (15.3%)	
Triple-negative	20 (15.2%)	114 (21.5%)	

Abbreviations: mtDNA, mitochondrial DNA; HR, Hormone receptor; HER2, human epidermal growth factor-2

The global likelihood ratio test for the full Cox regression model (including copy number and all prognostic factors) showed that all factors together significantly affect breast cancer prognosis(P<0.0001). Cox regression analyses model 1 in Table 3 shows that a higher mtDNA copy number (quintile 2–5) was significantly associated with a 1.476-fold higher risk of 5-year BC-specific recurrence (HR = 1.476[95% CI 1.073–2.031], *P* = 0.016), 1.418-fold higher risk of 5-year distant recurrence (HR = 1.412[95% CI 1.015–1.981], *P* = 0.040), and 1.522-fold higher risk of 5-year overall death (HR = 1.522[95% CI 1.011–2.291], *P* = 0.044). However, after adjusting for age at BC diagnosis, tumor size, lymph node involvement, grade, hormone receptor status, and HER2 status, only association with iDFS remained significant. In model 3, quintile 2–5 was significantly associated with shorter iDFS (5-year iDFS: adjusted HR = 1.433[95% CI 1.038–1.978], *P* = 0.028; Table 3).

**Table 3:** Results of Cox model on breast cancer risk for the quintile 2 to 5 of mtDNA copy number versus quintile 1 (as reference category)

	5-Year iDFS		5-Year DDFS		5-Year BCSS		5-Year OS	
	Quintile 2–5 versus quintile 1		Quintile 2–5 versus quintile 1		Quintile 2–5 versus quintile 1		Quintile 2–5 versus quintile 1	
Adjust model^*^	HR (95%CI)	*P*	HR (95%CI)	*P*	HR (95%CI)	*P*	HR (95%CI)	*P*
Model1	1.476 (1.073–2.031)	0.016	1.418 (1.015–1.981)	0.040	1.477 (0.980–2.226)	0.062	1.522 (1.011–2.291)	0.044
Model2	1.431 (1.040–1.970)	0.028	1.355 (0.970–1.894)	0.075	1.378 (0.914–2.078)	0.126	1.420 (0.942–2.140)	0.094
Model3	1.433 (1.038–1.978)	0.028	1.355 (0.966-1.900)	0.078	1.406 (0.928–2.130)	0.108	1.440 (0.951–2.179)	0.084

Abbreviations: iDFS, invasive disease-free survival; DDFS, distant disease-free survival; BCSS, breast cancer special survival; OS, overall survival; CI, confidence interval; mtDNA, mitochondrial DNA; HRs: hazard ratios

^*^ Model 1: adjusted for age at diagnosis; Model 2: same as model 1, plus hormone receptor status. HER2 status; Model 3: same as model 2, plus tumor size, Lymph node involvement, grade

### Stratification and interaction analysis

The associations between mtDNA copy number and BC survival were then evaluated by stratified analysis of age at diagnosis, tumor size, lymph node involvement, grade, hormone-receptor status, and HER2 status, while interaction analysis was also performed between these covariates and mtDNAcn on BC survival. Statistically significant multiplicative interactions on BC survival were found between mtDNA copy number and hormone-receptor status (adjusted p for interaction: 5-year BCSS: 0.028, 5-year OS: 0.022).

### Prognostic analysis in HR + patients

As shown in Table 4, we found that higher mtDNA copy numbers (quintile 2–5) were associated with shorter survival of the patients who were positive hormone-receptor status (HR+: 5-year BCSS: aHR = 2.340[95% CI 1.163–4.708], *P* = 0.017 and 5-year OS: aHR = 2.446[95% CI 1.218–4.913], *P* = 0.011). However, compared with HR-positive patients, the performance of mitochondrial copy number in HR-negative patients is the opposite and has no significance (Table 4). We further conducted a stepwise Cox proportional hazard analysis to evaluate the effects of clinical variables and mtDNA copy number on BCSS and OS in HR-positive patients. Three variables (node status, grade, mtDNAcn) were selected for the final regression model of BC-specific survival, while four variables (node status, mtDNAcn, grade, HER2) were selected for the final regression model of overall survival (Table 5). In the HR-positive subgroup final model, the mtDNAcn was an independent risk factor for 5-year BCSS with a 2.240-fold (95% CI, 1.204–4.863) increased risk for 5-year OS with a 2.464-fold (95% CI, 1.227–4.948) increased risk.

**Table 4:** Hazard ratio of tumor survival for all 661 patients with breast cancer according to the mtDNA copy number in different subgroups stratified by clinical parameters

Variables		5-Year iDFS			5-Year DDFS			5-Year BCSS			5-Year OS		
		Ajusted HR^*^(95%CI)	*P*	*P* for interaction	Ajusted HR^*^(95%CI)	*P*	*P* for interaction	Ajusted HR^*^(95%CI)	*P*	*P* for interaction	Ajusted HR^*^(95%CI)	*P*	*P* for interaction
Age				0.266			0.275			0.204			0.238
≤ 40	207	1.847 (1.047–3.259)	0.034		1.810 (0.982–3.336)	0.057		2.096 (0.946–4.645)	0.068		2.096 (0.946–4.645)	0.068	
>40	454	1.262 (0.853–1.869)	0.244		1.178 (0.784–1.769)	0.430		1.204 (0.738–1.964)	0.457		1.248 (0.767–2.031)	0.373	
HR				0.687			0.334			0.028			0.022
Negative	235	1.390 (0.847–2.282)	0.193		1.177 (0.714–1.939)	0.522		0.988 (0.586–1.667)	0.964		0.988 (0.586–1.665)	0.963	
Positive	426	1.473 (0.962–2.256)	0.075		1.520 (0.958–2.411)	0.075		2.340 (1.163–4.708)	0.017		2.446 (1.218–4.913)	0.011	
HER2				0.501			0.232			0.640			0.604
Negative	467	1.551 (1.018–2.362)	0.041		1.573 (0.997–2.482)	0.051		1.497 (0.860–2.606)	0.153		1.535 (0.884–2.667)	0.128	
Positive	194	1.336 (0.808–2.208)	0.258		1.135 (0.684–1.884)	0.623		1.287 (0.684–2.420)	0.434		1.318 (0.702–2.475)	0.390	
Grade				0.795			0.828			0.709			0.701
I + II	500	1.374 (0.954–1.981)	0.088		1.355 (0.920–1.994)	0.124		1.427 (0.887–2.295)	0.142		1.467 (0.914–2.356)	0.112	
III	161	1.596 (0.793–3.210)	0.190		1.290 (0.638–2.610)	0.478		1.347 (0.567-3.200)	0.499		1.375 (0.580–3.262)	0.469	
Tumor sizes				0.347			0.329			0.942			0.975
≤ 2	211	2.053 (0.997–4.230)	0.051		2.042 (0.946–4.405)	0.068		1.519 (0.657–3.508)	0.328		1.519 (0.657–3.508)	0.328	
>2	450	1.302 (0.907–1.868)	0.152		1.229 (0.843–1.793)	0.283		1.430 (0.885–2.312)	0.144		1.479 (0.916–2.387)	0.109	
Nodal status				0.170			0.240			0.208			0.290
Negative	255	0.941 (0.502–1.767)	0.851		0.882 (0.442–1.759)	0.721		0.766 (0.310–1.892)	0.564		0.864 (0.354–2.107)	0.747	
Positive	406	1.603 (1.101–2.334)	0.013		1.499 (1.016–2.211)	0.041		1.577 (0.986–2.522)	0.057		1.597 (0.999–2.553)	0.050	

Abbreviations: iDFS, invasive disease-free survival; DDFS, distant disease-free survival; BCSS, breast cancer special survival; OS, overall survival; CI, confidence interval; mtDNA, mitochondrial DNA; aHR: adjusted hazard ratio

^*^ adjusted for age at diagnosis, tumor size, lymph node involvement, grade, hormone receptor status, and HER2 status, except for the interaction factor

**Table 5:** Results of stepwise Cox regression analysis on breast cancer-specific survival and overall survival in HR-positive breast cancer patients

Factor (in order of entry)	HR (95%CI)	*P* (in final modal)
5-Year Breast Cancer-Specific Survival		
1. Nodal status(positive vs. negative)	4.148 (2.186–7.783)	<0.001
2. Grade(III vs. I + II)	1.859 (1.138–3.035)	0.013
3. mtDNAcn(Quintile 2–5 vs. Quintile 1)	2.420 (1.204–4.863)	0.013
5-Year Overall Survival		
1. Nodal status(positive vs. negative)	3.454 (1.900-6.279)	<0.001
2. mtDNAcn(Quintile 2–5 vs. Quintile 1)	2.464 (1.227–4.948)	0.011
3. Grade(III vs. I + II)	1.705 (1.046–2.777)	0.032
4. HER2(positive vs. negative)	1.537 (0.959–2.463)	0.073

## Discussion

This study evaluated the possible relation between mtDNA copy number in peripheral blood and BC clinical outcome in a cohort of 661 EBC cases. We found that patients with high mtDNA copy numbers showed significantly shorter iDFS than those with low mtDNA (5-year iDFS: adjusted HR = 1.433[95% CI 1.038–1.978], *P* = 0.028). The most significant and novel result of this study is that, in the HR + subgroup, patients with high copy number have a worse prognosis than those with low mtDNA (HR+: 5-year BCSS: aHR = 2.340[95% CI 1.163–4.708], *P* = 0.017 and 5-year OS: aHR = 2.446[95% CI 1.218–4.913], *P* = 0.011). In short, the high mtDNA copy number in peripheral blood led to a worse prognosis for EBC patients, especially in the HR-positive subgroup patients.

Previous studies have proposed that high leukocyte mtDNA copy number in patients may impair the immune defense against tumor cells and lead to poor prognosis. MtDNA content is regulated by a wide range of environmental and genetic factors. For example, raised mtDNA content has altered oxidative stress, aging, immune response activation, and environmental exposures [30–32]. Several studies have demonstrated that high leukocyte mtDNA content is associated with a worse prognosis for patients with glioma [22], HCC [21], and colorectal cancer [20]. These studies have reported that cancer patients with high leukocyte mtDNAcn may be in an immunosuppressive state. Furthermore, mitochondrial ROS, generally increased with mtDNA copy number, can inhibit the expansion and functions of helper T cells and killer cells in peripheral blood [33, 34]. In line with these findings, our study demonstrated that a higher leukocyte mtDNA copy number was associated with poor prognosis in HR-positive EBC patients.

However, several reports showed that lower mtDNAcn in breast tumor tissues was associated with a worse prognosis [10, 35, 36]. Only one previous study evaluated the progression role of mtDNA copy number in peripheral blood leucocytes in BC patients. Xia et al. reported that leukocyte mtDNA content is associated with the T stage of BC, indicating that high mtDNA content may facilitate cancer progression [23]. Nevertheless, the relationship between tumor size and prognosis is unstable, and Xia et al. did not have any follow-up data. We are the first and only study to explore the relationship between the mitochondrial DNA copy number of peripheral blood cells and the prognosis of BC patients. We mainly found that higher leukocyte mtDNA content was associated with poor BCSS and OS in HR-positive EBC patients.

In contrast to mtDNAcn reduction in breast tumor tissues, the studies of leukocyte mtDNAcn of BC patients and its association with prognosis have been heterogeneous. Rai et al. showed an inverse relation in blood vs. tissue samples in BC patients [37]. Our results were consistent with a meta-analysis that summarized the results of 18 studies involving 3961 cancer patients and concluded that elevated mtDNAcns in peripheral blood leukocytes and tumor tissues predict the opposite outcome of cancer [38]. Biologically, mtDNAcn is specific to different tissue types, reflecting differing energy requirements [39, 40]. Collectively, these findings support the point of view that changes in mtDNA content are involved in the progression of BC.

High mtDNA content was significantly associated with poor prognosis, mainly in the HR-positive subgroup but not in the HR-negative subgroup, indicating host characteristics’ modulating effects through unknown mechanisms. HR positivity was either ER + and/or PR+. After binding to estrogen or progesterone, HR can interact with the mtDNA to maintain mitochondrial function, inhibit cell apoptosis, and overcome oxidative damage [41, 42]. The different performance of mtDNA copy numbers in the HR + and HR-subgroups may be due to this interaction. Gao et al. found that estrogen indirectly regulated mitochondrial DNA replication and repair functions [43, 44]. Previous studies have identified that the instability of mtDNA due to exposure to estrogen induces cancer cell metastasis [45]. As a result, we proposed that in HR-positive patients, the oxidative damage in the tumor caused by estrogen or progesterone and the immunosuppression of the high copy number in the peripheral blood cells together lead to a significantly worse prognosis.

As the first exploration of a possible relationship between leukocyte mtDNAcn and survival in EBC patients, our study has several strengths, including long-term follow-up, more cases than previous studies, and comprehensive clinical data. There are several limitations to our study. First, as all patients were of Chinese origin, it is unclear whether our findings are Chinese Han population-specific or common in other populations. Second, we only performed association analyses between mtDNA content and the prognosis of BC patients. The underlying mechanisms through which mtDNA content affects immune functions need further investigation. Lastly, future correlation analysis of mtDNAcn in different tissues (e.g., leukocytes vs. tumor tissues) would help us better understand the relationship between mitochondrial biology and carcinogenesis.

## Conclusions

For the first time, we proposed that the prognostic value of leukocyte mtDNA content in EBC patients may depend on intrinsic tumor subtypes. Our study reminds us that we should pay special attention to HR-positive BC patients with high peripheral blood mitochondrial copy numbers when treating them because the prognosis of HR-positive BC patients may be worse. Once confirmed, leukocyte mtDNA content can serve as a useful biomarker to improve the prognosis prediction for BC patients.

## Electronic supplementary material

Below is the link to the electronic supplementary material.

Supplementary Material 1

Supplementary Material 2

Supplementary Material 3

Supplementary Material 4

## Data Availability

The data that support the findings of this study are available from Fujian Medical University Union Hospital, but restrictions apply to the availability of these data, which were used under license for the current study, and so are not publicly available. However, data are available from the authors upon reasonable request and with permission of Fujian Medical University Union Hospital. Fangmeng Fu, the corresponding author, should be contacted if someone wants to request the data from this study.

## Abbreviations

mtDNAcn: mitochondrial DNA copy number
BC: breast cancer
EBC: early-stage breast cancer
iDFS: invasive disease-free survival
DDFS: distant disease-free survival
BCSS: BC special survival
OS: overall survival
HR: hormone receptor
ROS: reactive oxygen species
AJCC: American Joint Commission on Cancer
TNM: tumor-node-metastasis
ER: estrogen receptor
PR: progesterone receptor
HER2: Human epidermal growth factor-2
HRs: hazard ratios
CI: confidence interval
aHR: adjusted hazard ratio
