# Supplementary material for: High leukocyte mitochondrial DNA copy number contributes to poor prognosis in breast cancer patients
Source: BMC Cancer. 2023 Apr 25;23:377. doi: 10.1186/s12885-023-10838-x (PMC10131463; doi:10.1186/s12885-023-10838-x)
Supplement: Supplementary file 1 — Supplementary Material 1 [file 12885_2023_10838_MOESM1_ESM.docx]

| **Probe** | **Chromosome** | **Location**  **(ref 37 database)** | **Amplification length**  **(sample, competitive)^*^** | **Primer binding region 1** | **Primer binding region 2** |
| --- | --- | --- | --- | --- | --- |
| ND1 | ChrMT | 3818-3959 | 170 (+0,-2) | CACCTCTGATTACTCCTGCCATCA | CAACATCGAATACGCCGCAG |
| reference segment 2p | Chr2 | 84500611-84500685 | 103 (+0,-2) | TGAGCCAAAAATTCAGAATACAAGGA | GTTTGCCTGCCTTCCAAGCAA |
| reference segment 10p | Chr10 | 31120531-31120675 | 173 (+0,-2) | CACTGAGCCCCAGAGACCTGAC | GTTTTCCCTGGAGGTGTGCATT |
| reference segment 16p | Chr16 | 25258129-25258426 | 326 (+0,-2) | TCCTCCACCAAGCTGATGTGTT | CTATTTCGGGGACAGGCCTGAA |
| reference segment 18p | Chr18 | 13438305-13438407 | 131 (+0,-2) | CAGAACGACTGTGGGGACAACA | AAGTCTCTCCTCCCACCTGGGT |
| reference segment 19p | Chr19 | 18958265-18958632 | 396 (+0,-2) | GGACTAGAATGGCCCTGGAGGA | TTCTGCAACGGACGTGGAAATA |
| reference segment 20p | Chr20 | 35865921-35866142 | 250 (+0,-2) | AGGGTGCTGGGATCAGAGAGAG | CATTTTGCCACCCTCCAGTAGC |

**Table S1 The information of ND1 or reference gene location and the sequence of specific probe combining area.**

^*^Sample: Sample DNA; Competitive: Competitive DNA.
