# Supplementary material for: High leukocyte mitochondrial DNA copy number contributes to poor prognosis in breast cancer patients
Source: BMC Cancer. 2023 Apr 25;23:377. doi: 10.1186/s12885-023-10838-x (PMC10131463; doi:10.1186/s12885-023-10838-x)
Supplement: Supplementary file 2 — Supplementary Material 2 [file 12885_2023_10838_MOESM2_ESM.docx]

**Table S3 Treatment characteristics of patients**

| **Treatment** | **No. of patients** | **Percent** |
| --- | --- | --- |
|  | **(n=661)** | **%** |
| **Chemotherapy** |  |  |
| **Anthracyclines** | **96** | **14.5** |
| **Taxanes** | **84** | **12.7** |
| **Anthracyclines and taxanes** | **431** | **65.2** |
| **Others** | **19** | **2.9** |
| **Without chemotherapy** | **29** | **4.4** |
| **Missing** | **2** | **0.3** |
| **Endocrine therapy** |  |  |
| **Yes** | **417** | **63.1** |
| **No** | **244** | **36.9** |
| **Targeted therapy** |  |  |
| **Yes** | **109** | **16.5** |
| **No** | **551** | **83.3** |
| **Unknown** | **1** | **0.2** |
| **Radio therapy** |  |  |
| **Yes** | **324** | **49.0** |
| **No** | **335** | **50.7** |
| **Unknown** | **2** | **0.3** |

Others：anthracycline only; and cyclophosphamide, methotrexate, or fluorouracil only
