# Supplementary material for: High leukocyte mitochondrial DNA copy number contributes to poor prognosis in breast cancer patients
Source: BMC Cancer. 2023 Apr 25;23:377. doi: 10.1186/s12885-023-10838-x (PMC10131463; doi:10.1186/s12885-023-10838-x)
Supplement: Supplementary file 3 — Supplementary Material 3 [file 12885_2023_10838_MOESM3_ESM.docx]

**Table S2 Results of Cox model on survival for the quintilesof mtDNAcopy number versus quintile1 (as reference category)**

|  |  | **5-Year iDFS** | |  | **5-Year DDFS** |  | **5-Year BCSS** |  | **5-Year OS** |  |
| --- | --- | --- | --- | --- | --- | --- | --- | --- | --- | --- |
|  |  | **Quintile 2/3/4/5 versus Quintile 1** | | | **Quintile 2/3/4/5 versus Quintile 1** | | **Quintile 2/3/4/5 versus Quintile 1** | | **Quintile 2/3/4/5 versus Quintile 1** | |
| **Adjust model^*^** |  | **HR (95%CI)** | ***P*** | | **HR (95%CI)** | ***P*** | **HR (95%CI)** | ***P*** | **HR (95%CI)** | ***P*** |
| Model1 | Quintile 1 | Reference |  | | Reference |  | Reference |  | Reference |  |
|  | Quintile 2 | 1.637 (1.116-2.399) | 0.012 | | 1.561 (1.047-2.329) | 0.029 | 1.438 (0.876-2.362) | 0.151 | 1.482 (0.905-2.427) | 0.118 |
|  | Quintile 3 | 1.460 (0.987-2.160) | 0.058 | | 1.357 (0.899-2.049) | 0.146 | 1.452 (0.880-2.393) | 0.144 | 1.492 (0.907-2.452) | 0.115 |
|  | Quintile 4 | 1.656 (1.129-2.429) | 0.010 | | 1.622 (1.087-2.421) | 0.018 | 1.730 (1.069-2.801) | 0.026 | 1.768 (1.094-2.857) | 0.019 |
|  | Quintile 5 | 1.191 (0.798-1.778) | 0.392 | | 1.158 (0.760-1.765) | 0.494 | 1.299 (0.781-2.160) | 0.313 | 1.348 (0.813-2.234) | 0.246 |
| Model2 | Quintile 1 | Reference |  | | Reference |  | Reference |  | Reference |  |
|  | Quintile 2 | 1.507 (1.027-2.212) | 0.036 | | 1.438 (0.963-2.147) | 0.076 | 1.267 (0.771-2.084) | 0.350 | 1.309 (0.798-2.146) | 0.286 |
|  | Quintile 3 | 1.467 (0.991-2.172) | 0.056 | | 1.340 (0.887-2.024) | 0.165 | 1.388 (0.841-2.292) | 0.199 | 1.432 (0.870-2.357) | 0.157 |
|  | Quintile 4 | 1.631 (1.112-2.393) | 0.012 | | 1.548 (1.037-2.311) | 0.033 | 1.631 (1.006-2.644) | 0.047 | 1.666 (1.030-2.694) | 0.038 |
|  | Quintile 5 | 1.155 (0.773-1.725) | 0.481 | | 1.116 (0.732-1.700) | 0.610 | 1.243 (0.747-2.067) | 0.403 | 1.288 (0.777-2.136) | 0.326 |
| Model3 | Quintile 1 | Reference |  | | Reference |  | Reference |  | Reference |  |
|  | Quintile 2 | 1.575 (1.069-2.321) | 0.022 | | 1.513 (1.009-2.271) | 0.045 | 1.337 (0.809-2.210) | 0.258 | 1.371 (0.832-2.261) | 0.215 |
|  | Quintile 3 | 1.388 (0.935-2.061) | 0.104 | | 1.255 (0.827-1.903) | 0.286 | 1.335 (0.804-2.216) | 0.265 | 1.363 (0.824-2.256) | 0.228 |
|  | Quintile 4 | 1.614 (1.098-2.372) | 0.015 | | 1.519 (1.015-2.273) | 0.042 | 1.596 (0.981-2.599) | 0.059 | 1.620 (0.997-2.630) | 0.051 |
|  | Quintile 5 | 1.187 (0.793-1.777) | 0.404 | | 1.155 (0.756-1.765) | 0.504 | 1.354 (0.812-2.259) | 0.246 | 1.401 (0.843-2.330) | 0.194 |

Abbreviations: iDFS, invasive disease-free survival; DDFS, distant disease-free survival; BCSS, breast cancer special survival; OS, overall survival; CI, confidence interval; mtDNA, mitochondrial DNA; HR, hazard ratio.

^*^Model 1: adjusted for age at diagnosis; Model 2: same as model 1, plus hormone receptor status. HER2 status; Model 3: same as model 2, plus tumor size, Lymph node involvement, grade.
