# Supplementary material for: High leukocyte mitochondrial DNA copy number contributes to poor prognosis in breast cancer patients
Source: BMC Cancer. 2023 Apr 25;23:377. doi: 10.1186/s12885-023-10838-x (PMC10131463; doi:10.1186/s12885-023-10838-x)
Supplement: Supplementary file 4 — Supplementary Material 4 [file 12885_2023_10838_MOESM4_ESM.docx]

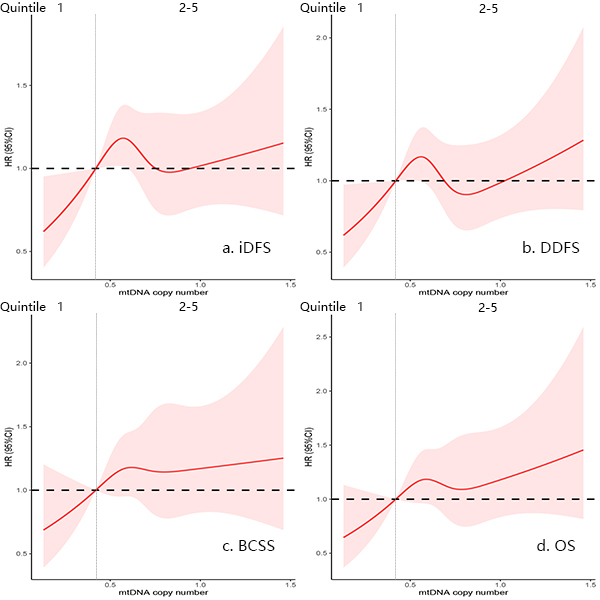


**Figure S1.** Adjusted nonlinear splines (and 95% confidence bands) for the association between increasing mitochondrial (mt) DNA copy number and hazard ratio (HR) of iDFS (a), DDFS (b), BCSS (c), OS(d) (adjusted for age at diagnosis, tumor size, Lymph node involvement, grade, hormone receptor status, and HER2 status). Vertical dotted lines indicate the threshold for the first quintile of mtDNA copy number; the first quintile value of mtDNA is set as a reference (HR = 1; horizontal dashed line).
